# OGP: A Repository of Experimentally Characterized *O*-glycoproteins to Facilitate Studies on *O*-glycosylation

**DOI:** 10.1016/j.gpb.2020.05.003

**Published:** 2021-02-10

**Authors:** Jiangming Huang, Mengxi Wu, Yang Zhang, Siyuan Kong, Mingqi Liu, Biyun Jiang, Pengyuan Yang, Weiqian Cao

**Affiliations:** 1 Department of Chemistry and Institutes of Biomedical Sciences, Fudan University, Shanghai 200032, China; 2 The Fifth People’s Hospital, Fudan University, and the Shanghai Key Laboratory of Medical Epigenetics, the International Co-laboratory of Medical Epigenetics and Metabolism, Ministry of Science and Technology, Fudan University, Shanghai 200032, China; 3 NHC Key Laboratory of Glycoconjugates Research (Fudan University), Shanghai 200032, China

**Keywords:** *O*-glycosylation, *O*-glycoprotein repository, Site prediction, *O*-glycoprotein related website, Data mining

## Abstract

Numerous studies on cancers, biopharmaceuticals, and clinical trials have necessitated comprehensive and precise analysis of protein ***O*-glycosylation**. However, the lack of updated and convenient databases deters the storage of and reference to emerging *O*-glycoprotein data. To resolve this issue, an ***O*-glycoprotein repository** named OGP was established in this work. It was constructed with a collection of *O*-glycoprotein data from different sources. OGP contains 9354 *O*-glycosylation sites and 11,633 site-specific *O*-glycans mapping to 2133 *O*-glycoproteins, and it is the largest *O*-glycoprotein repository thus far. Based on the recorded *O*-glycosylation sites, an *O*-glycosylation **site prediction** tool was developed. Moreover, an OGP-based website is already available (https://www.oglyp.org/). The website comprises four specially designed and user-friendly modules: statistical analysis, database search, site prediction, and data submission. The first version of OGP repository and the website allow users to obtain various *O*-glycoprotein-related information, such as protein accession Nos., *O*-glycosylation sites, *O*-glycopeptide sequences, site-specific *O*-glycan structures, experimental methods, and potential *O*-glycosylation sites. *O*-glycosylation **data mining** can be performed efficiently on this website, which will greatly facilitate related studies. In addition, the database is accessible from OGP website (https://www.oglyp.org/download.php).

## Introduction

Comprehensive and precise analysis of *O*-glycoproteins would potentially further the current understanding of their roles in many physiological and pathological phenomena, such as intercellular communication [1], hereditary disorders, immune deficiencies, and cancers [2], [3], [4]. Great efforts have been made to analyze the complexity of *O*-glycosylation. Recent technological advancements in many fields, especially in mass spectrometry (MS), lead to impressive data on *O*-glycoproteins [5], [6], [7], [8], [9], [10], [11], [12], [13], [14]. However, the lack of up-to-date and curated databases hinders the archive, query, and utilization of emerging *O*-glycoprotein data.

Numerous studies have attempted to develop glycosylation-related databases [15], [16], [17], [18], [19], [20], [21], [22], [23], [24], [25], [26], [27], [28]. However, most of these databases are focused on *N*-glycoproteins. Only a few databases contain data on *O*-glycoproteins. The most extensively used repository, UniCarbKB [16], provides massive *N*-glycoprotein data and limited *O*-glycoprotein records. The dbPTM [18], [19] is an integrated resource containing over 130 types of post-translational modifications (PTMs). However, it does not provide information regarding site-specific *O*-glycosylation. O-GLYCBASE [15] provides information regarding both glycans and glycosylation sites and is the most widely used database in *O*-glycosylation studies. Nevertheless, it has not been updated since 2002. Besides, it contains merely 189*O*-glycoproteins and 2142*O*-glycosylation sites, lagging behind current *O*-glycoproteomic data. In short, current *O*-glycoprotein databases are less satisfactory with notable issues, including insufficient records, unknown data confidence, outdated data, and user-unfriendly interface (Table S1).

It can be said that the dearth of *O*-glycoprotein databases has greatly impeded the development of the *O*-glycosylation study. Recently, large-scale analyses of *O*-glycosylation sites and intact *O*-glycopeptides have gradually become possible. For example, Steentoft et al. [5] exploited a glyco-engineering method termed “SimpleCell” for large-scale identification of *O*-glycosylation sites. Yang et al. [7] developed a method called “EXoO” for large-scale analysis of intact *O*-glycopeptides. However, functional studies on *O*-glycoproteins are yet limited. In addition to the complexity of *O*-glycosylation, another primary factor limiting studies on *O*-glycosylation is the difficulty in retrieving information from large data to select candidate *O*-glycoproteins. Thus, an updated *O*-glycosylation database providing curated information of protein *O*-glycosylation status, site-specific *O*-glycans, analytical methods, and other related information is required and would accelerate studies on *O*-glycosylation.

In this study, an *O*-glycoprotein repository named OGP was constructed. OGP contains 9354*O*-glycosylation sites and 11,633 site-specific *O*-glycans mapping to 2133*O*-glycoproteins. To our knowledge, OGP is the most comprehensive repository for experimentally characterized *O*-glycoproteins thus far. An *O*-glycosylation site prediction tool was also developed on the basis of the recorded sites. An OGP-based website was well established (http://www.oglyp.org/) to facilitate access to the database. The website contains four modules: statistical analysis, database search, site prediction, and data submission. All the aforementioned *O*-glycoprotein data can be easily obtained on the website. Such a comprehensive, user-friendly, and open-access *O*-glycoprotein repository would greatly benefit researches on *O*-glycosylation, development of *O*-glycoprotein drugs, and clinical studies.

## Construction of the OGP repository

**Figure 1:**
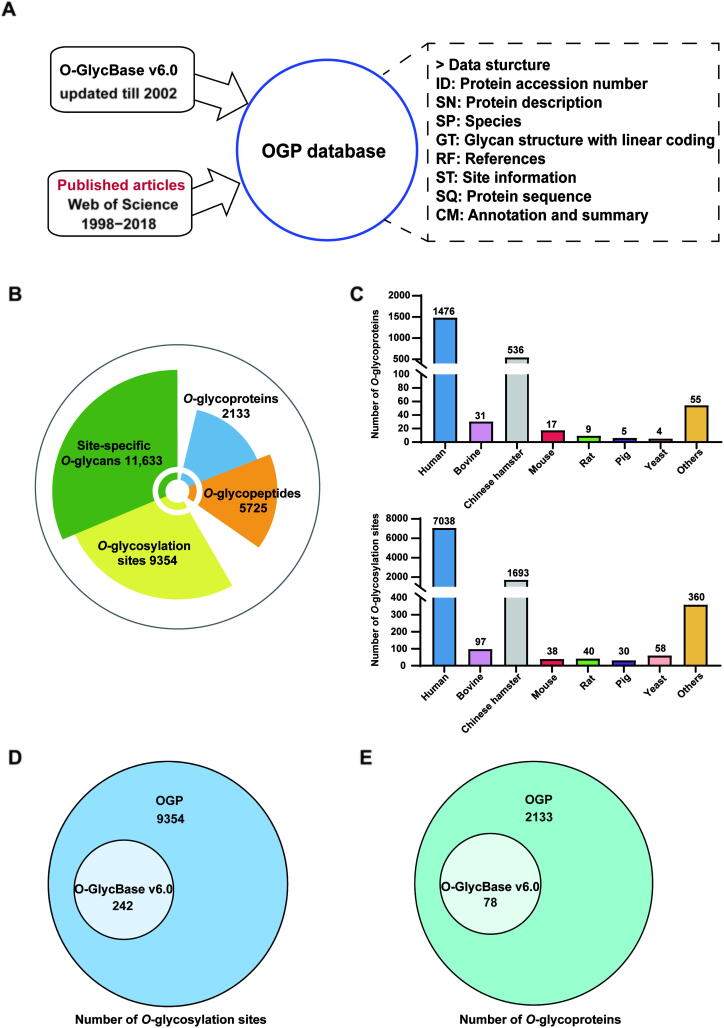
**Overview of the OGP repository. A.** OGP data collection. **B.** The scale of the OGP repository. **C.** Species distribution of *O*-glycoproteins and *O*-glycosylation sites in OGP. **D.** Comparison of OGP with O-GlycBase v6.0 on glycosylation site level. **E.** Comparison of OGP with O-GlycBase v6.0 on glycoprotein level.

The OGP knowledgebase was constructed by integrating experimentally verified *O*-glycoproteins reported between 1998 and 2018 and other existing *O*-glycoprotein databases [15] (Figure 1**A**). All proteins were manually curated, aligned with UniProt entries, and merged. Detailed methods of information extraction from literatures are described in File S1. In total, 9354 *O*-glycosylation sites and 11,633 site-specific *O*-glycans mapping to 2133 *O*-glycoproteins of different species have been recorded in the database (Figure 1B). The distribution of species in OGP shows that 69% (1476/2133) *O*-glycoproteins and 75% (7038/9354) *O*-glycosylation sites belong to *Homo sapiens* (Figure 1C), indicating a prevailing *O*-glycosylation study in *Homo sapiens*. The scale of the OGP repository is more than 20-fold bigger than the existing O-GlycBase v6.0 (Figure 1D and E). This database will also be updated periodically with newly published data in the future.

The database records data such as proteins, peptide sequences, *O*-glycosylation sites, and site-specific *O*-glycans. For each site and site-specific *O*-glycan, detailed experimental information, such as sample sources, digestion enzymes, enrichment methods, and analytical methods, is integrated. Besides, all *O*-glycoproteins recorded in the database have been aligned with their UniProt entries. Thus, additional data, including protein sequence annotation, subcellular location, and other PTMs, can be conveniently obtained. To better obtain topological information regarding *O*-glycans, a linear coding method (File S2) has been used in this database to record site-specific *O*-glycan structures. Furthermore, analytical strategies for each *O*-glycopeptide, such as immunoprecipitation, gel filtration, and MS methods, were manually extracted, verified, and recorded in the database. These data are easily retrievable from the OGP-based website.

## Development of an *O*-glycosylation site prediction model

**Figure 2:**
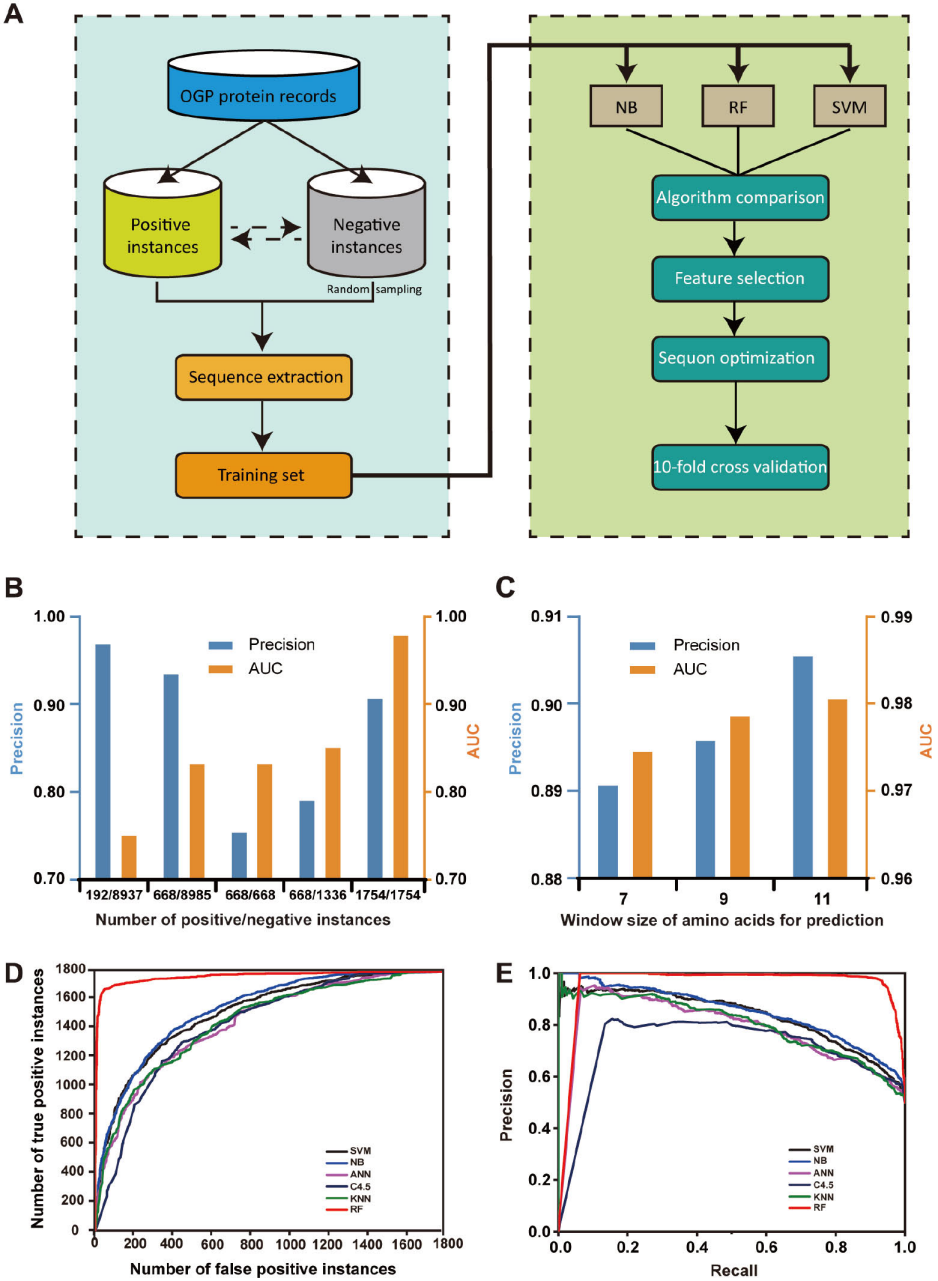
**Development of *O*-glycosylation site prediction model. A.** Workflow for building OGP-based *O*-glycosylation site prediction model. **B.** Effect of scales and ratios of positive and negative instances on model prediction performance. **C.** Influence of amino acid residue length on the performance of the site prediction model. **D.** ROC curves of each classification algorithm. **E.** Precision recall curves of each classification algorithm. NB, naïve Bayesian; RF, random forest; SVM, support vector machine; ROC, receiver operating characteristic; AUC, area under the ROC curve; ANN, artificial neural networks; C4.5, C4.5 decision tree; KNN, *k*-nearest neighbors.

Since *O*-glycosylation is highly complex but important, it is significant to better understand glycosylation patterns [29], [30], [31], [32]. As a meaningful trial, an *O*-glycosylation site prediction model was developed using *O*-glycosylation sites, which were meticulously selected from OGP database. The rule of the selection was that the sites must be identified by at least one solid method to confirm the reliability and unambiguousness. The site prediction model was generated through three primary steps (Figure 2**A**; File S3): 1) construction of a dedicated training set; 2) optimization of parameters; 3) evaluation of site prediction performance. Through systematic optimization, a dedicated training set was established with a 1:1 ratio of positive to negative instances (1754 positive site-central sequences and 1754 negative site sequences) (Figure 2B; File S3). Sequences with 11 amino acid residues were considered preferable (Figure 2C; File S3). Thereafter, the performance of different algorithms on *O*-glycosylation site prediction was compared using Weka 3.8 as a data mining tool. The random forest (RF) algorithm displayed the best performance (Figure 2D and E; File S3) and was used to construct the prediction model. Ten-fold cross validation indicated that the prediction model has high accuracy and sensitivity [area under the receiver operating characteristic curve (AUC) value = 0.983, precision value = 0.915, recall value = 0.909].

## Construction of the OGP-based website

**Figure 3:**
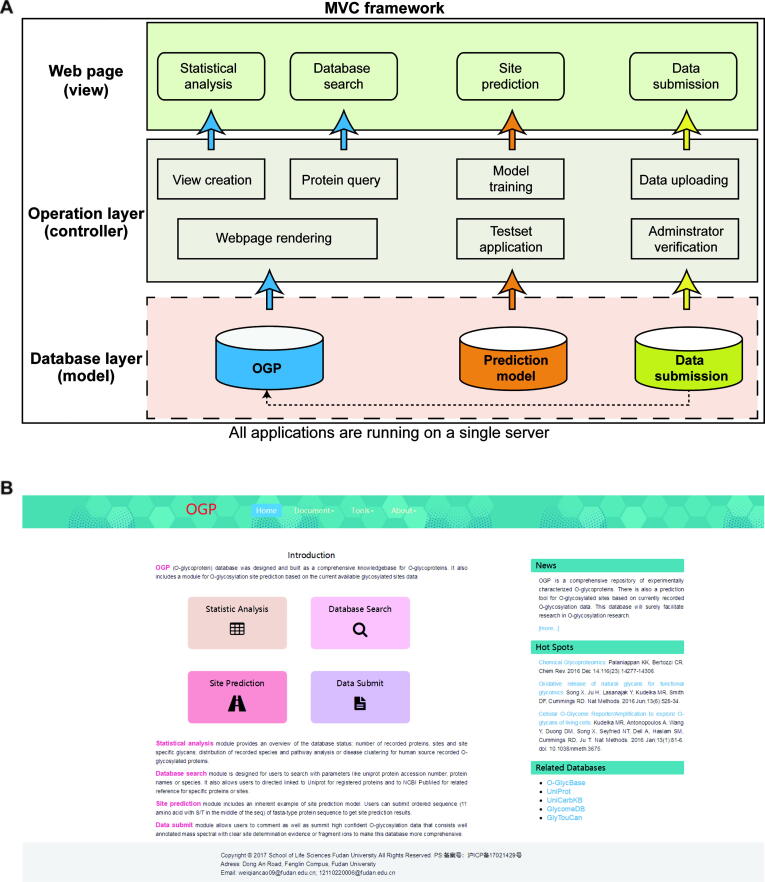
**Construction of OGP-based website. A.** The MVC framework of the OGP-based website. **B.** Homepage of the website. MVC, Model View Controller.

Based on the OGP database, a dedicated website was constructed using hypertext markup language (HTML), cascading style sheet (CSS), JavaScript (JS), and professional hypertext preprocessor (PHP). The design of the website is shown in Figure 3**A**. It contains three repositories in the underlying database layer: OGP, prediction model, and data submission. OGP repository is the core database that stores *O*-glycosylated protein sequences, sites, site-specific *O*-glycans, corresponding experimental data, and references. The prediction model contains a model file and an inherent training set. Data submission is designed to preserve user-uploaded information. By performing a set of actions including protein query, prediction model training, and data uploading in the operation layer, the website outputs four modules: statistical analysis, database search, site prediction, and data submission. The website is supported by most common web browsers such as Internet Explorer, Mozilla Firefox, Google Chrome, Safari, and Opera.

## Utility and the interface of the OGP website

The OGP-based website, equipped with a user-friendly graphical interface, is already available at http://www.oglyp.org/ and comprises four main modules: statistical analysis, database search, site prediction, and data submission. Furthermore, other functions, including database downloading, latest literature displaying, and useful database accesses (UniProt, UniCarbKB, and O-GlycBase), are also provided. The homepage of this website is shown in Figure 3B. Furthermore, the website provides detailed instructions and frequently asked questions (FAQ) to facilitate users.

**Supplementary figure S1:**
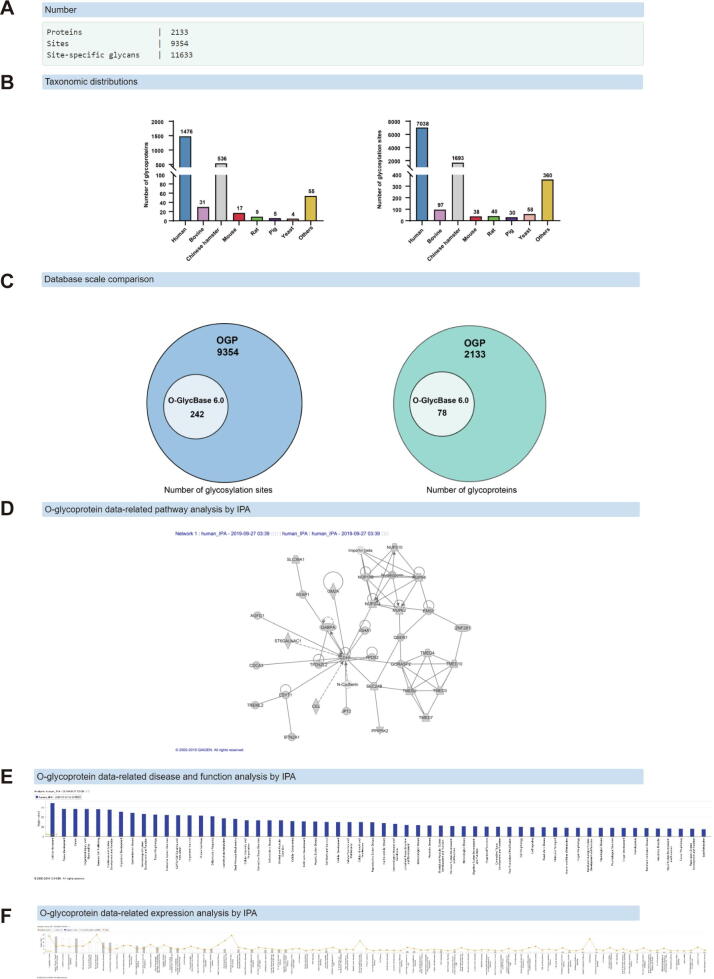
**Demonstration of statistical analysis module in OGP website A.** The scale of OGP database. **B.** Taxonomic distribution of O-glycoproteins and O-glycosylation sites in OGP database. **C.** Database scale comparison between the OGP and O-GlycBase v6.0 on glycosylation site level and glycoprotein level. **D.** O-glycoprotein data-related pathway analysis by IPA, ingenuity pathway analysis. **E.** O-glycoprotein data-related disease and function analysis by IPA. **F.** O-glycoprotein data-related expression analysis by IPA.

The “statistical analysis” module provides an overview of the OGP repository, including the scale of total *O*-glycoproteins, *O*-glycosylation sites, and site-specific *O*-glycans (Figure S1A), taxonomic distribution of *O*-glycoproteins and *O*-glycosylation sites (Figure S1B), database-scale comparison between OGP and O-GlycBase v6.0 (Figure S1C), *O*-glycoprotein data-related analyses by ingenuity pathway analysis (IPA) (Figure S1D–F). Furthermore, extra information can be fetched from this module. For example, more than 95% of the reported *O*-glycosylation sites are present in mammalians, 75% of which are present in *Homo sapiens*, indicating that *O*-glycosylation in other species warrants further analysis. All statistical information would be updated in real-time with the expansion of the OGP database.

**Figure 4:**
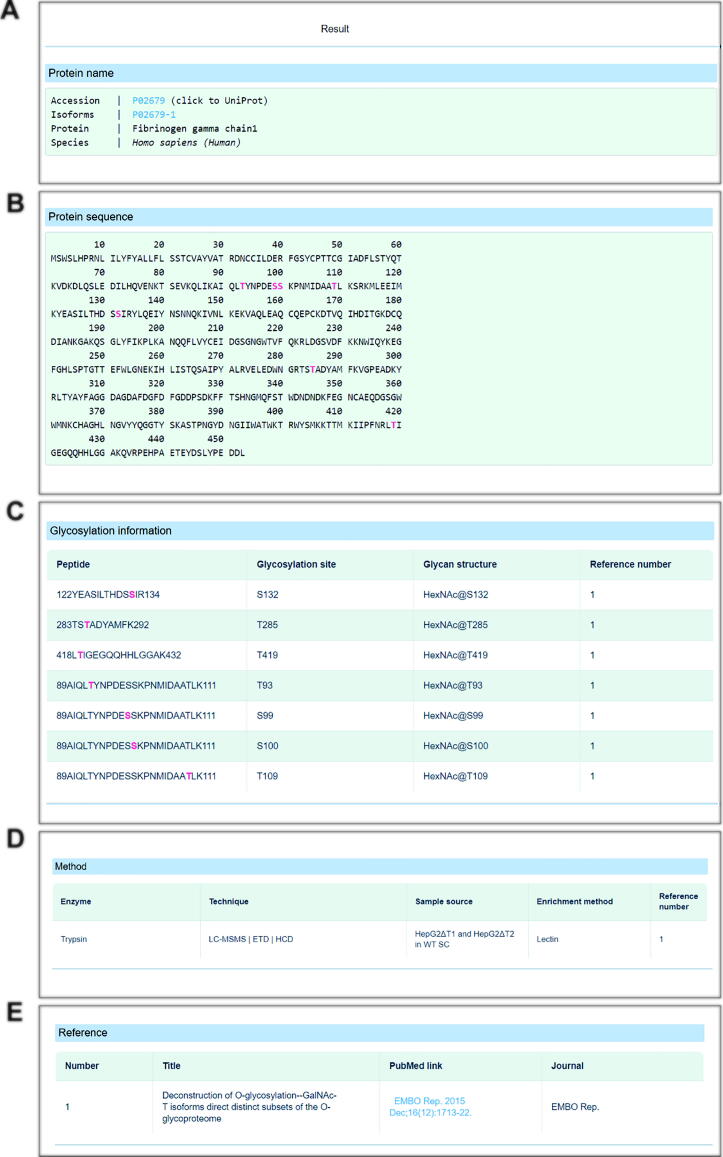
**A webpage returned from a query for Fibrinogen gamma chain. A.** Basic information of the *O*-glycoprotein. **B.** Protein sequence and all recorded *O*-glycosylation sites highlighted in pink. **C.** Experimentally verified *O*-glycopeptides and site-specific *O*-glycans. **D.** Corresponding experimental methods. **E.** Related source of references.

**Supplementary figure S2:**
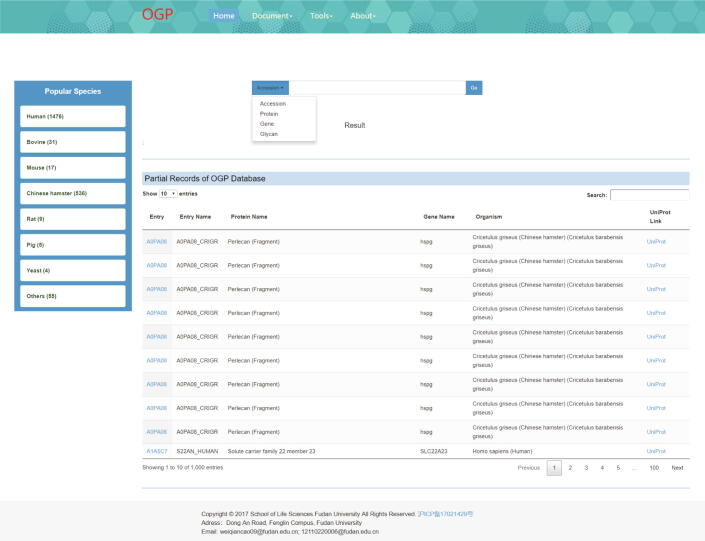
**The webpage of database search module**.

In the “database search” module, users can retrieve *O*-glycoproteins flexibly by specifying the gene name, protein name, UniProt accession No., or glycan structure (Figure S2). Figure 4 shows a webpage returned from a query of fibrinogen gamma chain (OGP database search accession No.: P02679). These results comprise well-structured data on protein *O*-glycosylation, including basic protein information (*i.e.*, protein name, UniProt accession No., and species, Figure 4A), protein sequences and all recorded *O*-glycosylation sites highlighted in pink (Figure 4B), all experimentally verified *O*-glycopeptides and site-specific *O*-glycans (Figure 4C), and corresponding experimental methods, identifiers, and source references (Figure 4D and E).

**Supplementary figure S3:**
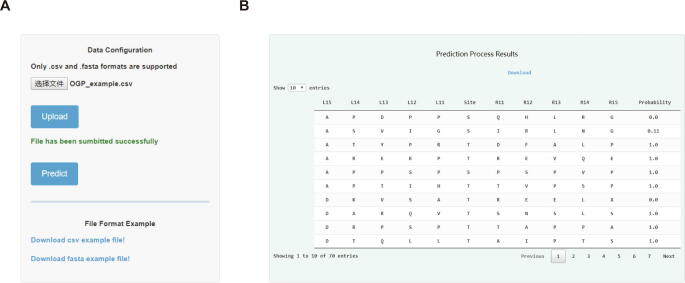
The webpage of site prediction module A. Submission of required prediction data. **B.** The prediction results for each site.

The site prediction model developed herein has also been incorporated into the website to enable *O*-glycosylation site prediction. As is shown in Figure S3A, users can either fill out the template file with aligned site-central sequences as instructed or simply upload a typical protein FASTA-format file and click on “predict”. The prediction results for each site can be then displayed directly on the right side of the webpage (Figure S3B). Prediction scores range between 0 and 1; scores higher than 0.5 indicate positive sites, while those less than or equal to 0.5 indicate a highly probably non-*O*-glycosylation site. The higher the score, the greater the probability of a site being *O*-glycosylated and *vice versa.* The results can also be downloaded, as shown in Figure S3B.

The “data submission” module enables users to upload new data into the OGP database or submit feedbacks. All the new submitted data and feedbacks are carefully recorded in a backend database and will be revised manually by experts at regular intervals. Both a template form and an online form are accepted during a submission. What’s more, when users upload the data by file, there will be a real-time feedback shown below to inform users of those *O*-glycoproteins already in OGP database.

In addition, the database is accessible from OGP website. Downloading pages can be found in the drop-down menu of tools on OGP homepage (https://www.oglyp.org/download.php). The detailed top 500 entries could be directly downloaded. Besides, there is a basic version of the database, which provides all the *O*-glycoprotein accessions and the corresponding *O*-glycosylation sites for users to download freely. The whole database could also be provided if users apply for it through E-mail request. The applying method is illustrated on the website (http://www.oglyp.org/download.php).

## Conclusion

The OGP repository, containing 9354*O*-glycosylation sites and 11,633 site-specific *O*-glycans mapping to 2133*O*-glycoproteins, is the most comprehensive *O*-glycoprotein repository thus far. All data contained in the OGP repository have been manually curated, and the proteins have been aligned with UniProt entries and merged. Based on recorded site data, an *O*-glycosylation site prediction tool has been developed to facilitate the prediction of *O*-glycosylation sites. The OGP-based website is available at http://www.oglyp.org/ and contains four specially designed, user-friendly, functional modules: statistical analysis, database search, site prediction, and data submission. The initial version of the OGP repository and OGP-based website provide various information on *O*-glycoproteins, such as protein accession Nos., *O*-glycopeptide sequences, site-specific *O*-glycan structures, experimental methods, and potential *O*-glycosylation sites. *O*-glycosylation data mining can be carried out efficiently using this website. The OGP repository would greatly facilitate studies on *O*-glycosylation. The scale and the content of this database are intended to be continuously expanded in subsequent versions of the OGP repository.

## Availability

OGP prediction tool is freely available at http://www.oglyp.org/predict.php. OGP database is freely available at http://www.oglyp.org/download.php.

Competing interests.

The authors have declared no competing interests.

### CRediT authorship contribution statement

**Jiangming Huang:** Methodology, Software, Resources, Data curation, Writing – original draft, Writing – review & editing. **Mengxi Wu:** Methodology, Resources, Data curation, Writing – original draft, Writing – review & editing, Visualization. **Yang Zhang:** Software. **Siyuan Kong:** Data curation. **Mingqi Liu:** Writing – review & editing. **Biyun Jiang:** Data curation. **Pengyuan Yang:** Supervision, Project administration, Funding acquisition. **Weiqian Cao:** Conceptualization, Methodology, Writing – original draft, Writing – review & editing, Supervision, Project administration, Funding acquisition.

## Supplementary material

Supplementary File S1 **Literature extraction**

Supplementary File S2 **Linear coding for glycan**

Supplementary File S3 **Construction of OGP-based site prediction model**
