# Supplementary material for: OGP: A Repository of Experimentally Characterized O-glycoproteins to Facilitate Studies on O-glycosylation
Source: Genomics Proteomics Bioinformatics. 2021 Feb 10;19(4):611–8. doi: 10.1016/j.gpb.2020.05.003 (PMC9039567; doi:10.1016/j.gpb.2020.05.003)
Supplement: Supplementary File S1 — Literature extraction [file mmc1.docx]

**File S1 Literature extraction**

Literatures published between 1998 and 2018 that matched searching key words of “*O*-glyc*” and “mass spect*” was retrieved from Web of Science core collection. A total of 1775 records were carefully reviewed manually. Relative strict criteria for *O*-glycopeptide selection were applied when screening: identification must include at least one solid validation method to ensure unambiguous identification of *O*-glycosylation; for example, sites validated by Edman sequencing [1] and HCD, ETD tandem MS [2]; glycan structures verified by lectin affinity chromatography [3] or sequential enzymatic release techniques [4] to fully elucidate the glycan structures. Identification with merely MS1 *m*/*z* deduction or single broad-specificity lectin affinity was discarded. Identified glycoprotein, glycosylation sites and corresponding glycan structures were then filtered and recorded. In addition, sample information like species (*i.e.*, human, mouse, and bovine), organs (*i.e.*, liver, kidney, and plasma), identification and verification techniques (*i.e.*, tandem MS, NMR, and lectin affinity chromatography) were recorded at length. Every entry recorded was manually checked and aligned with its UniProt information on protein accession number, protein name, sequences, and indexes of glycosylation site to guarantee explicit *O*-glycoprotein information. Links to UniProt were also provided on queried pages to assist cross-reference. What’s more, references for every single *O*-glycosylation site identification were detailed recorded in the form of article titles, PubMed-formatted citations, publication date, and links to PubMed webpages.

**References**

[1] Bousfield GR, Butnev VY, Butnev VY. Identification of twelve O-glycosylation sites in equine chorionic gonadotropin β and equine luteinizing hormone β by solid-phase Edman degradation. Biol Reprod 2001;64:136–47.

[2] Durham M, Regnier FE. Targeted glycoproteomics: serial lectin affinity chromatography in the selection of O-glycosylation sites on proteins from the human blood proteome. J Chromatogr A 2006;1132:165–73.

[3] Darula Z, Sarnyai F, Medzihradszky KF. O-glycosylation sites identified from mucin core-1 type glycopeptides from human serum. Glycoconj J 2016;33:435–45.

[4] Halfinger B, Sarg B, Lindner HH. Evaluation of non-reductive β-elimination/Michael addition for glycosylation site determination in mucin-like O-glycopeptides. Electrophoresis 2011;32:3546–53.
