# Supplementary material for: OGP: A Repository of Experimentally Characterized O-glycoproteins to Facilitate Studies on O-glycosylation
Source: Genomics Proteomics Bioinformatics. 2021 Feb 10;19(4):611–8. doi: 10.1016/j.gpb.2020.05.003 (PMC9039567; doi:10.1016/j.gpb.2020.05.003)
Supplement: Supplementary File S2 — Linear coding for glycan [file mmc2.docx]

**File S2 Linear coding for glycan**

*O*-glycans with branch information were transformed to unified formats of linear-coded structural forms [1], as depicted in Figure S4. Glycan units identified by MS were unified as HexNAc, Hex, Fuc, NeuAc, and NeuGc respectively. Branched structures were transformed to nested forms with baskets left adjacent to branched nodes. Linkages or bond information like alpha, beta conformation and/or 1,3-, 2,4- bond linkages, were also manually checked and recorded, although only a few of glycans have such information currently. Furthermore, glycans were assigned to corresponding sites with a connector “@”, followed by an abbreviated threonine (T), serine (S), or tyrosine (Y) and the index of the *O*-glycosylation site. For sites that have multiple glycans, all site-specific glycans were carefully collected, recorded, and separated using semicolons. As a result, variant glycan compositions on a single site can well demonstrate the overall status and the heterogeneity of *O*-glycosylation at present.


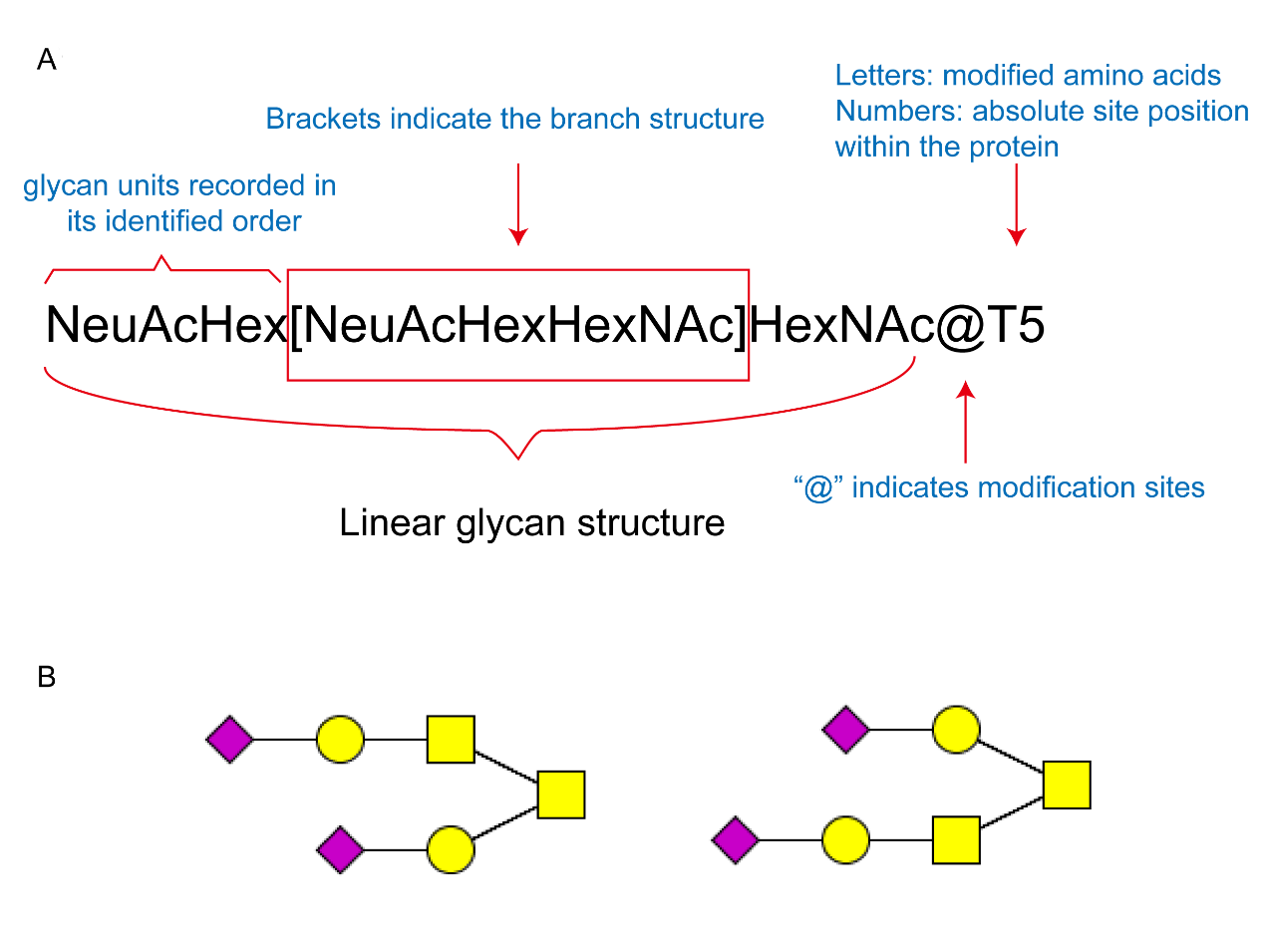


**Figure S4 The coding rule for glycan structure in OGP database**

**A.** Linear glycan structure recorded in OGP and instructions; **B.** Putative glycan structure of corresponding linear structure in panel A.

**References**

[1] Tsuchiya S, Yamada I, Aoki-Kinoshita KF. GlycanFormatConverter: a conversion tool for translating the complexities of glycans. Bioinformatics 2019;35:2434–40.
