# Supplementary material for: OGP: A Repository of Experimentally Characterized O-glycoproteins to Facilitate Studies on O-glycosylation
Source: Genomics Proteomics Bioinformatics. 2021 Feb 10;19(4):611–8. doi: 10.1016/j.gpb.2020.05.003 (PMC9039567; doi:10.1016/j.gpb.2020.05.003)
Supplement: Supplementary File S3 — Construction of OGP-based site prediction model [file mmc3.docx]

**File S3 Construction of OGP-based site prediction model**

The process of building OGP-based *O*-glycosylation site prediction model mainly contained three steps, as is shown in Figure 2A. Firstly, a dedicated training set was constructed with positive *O*-glycosylation sties and negative *O*-glycosylation sites. The positive *O*-glycosylation sites were manually extracted from experimentally identified and verified human *O*-glycosylated sites from OGP. Negative sites were generated by randomly sampling from the remaining sites of the corresponding *O*-glycoprotein (Step 1). Secondly, a series of parameter optimization process were conducted to improve the performance of the prediction model and to maximally avoid oversampling effects. The amino acid length as well as classification algorithms were carefully optimized (Step 2). Finally, evaluation of site prediction performance was conducted with 10-fold cross validation (Step 3) to further prove the accuracy and stability of this model.

**Optimization of positive/negative instances ratio**

The impact of positives and negatives proportion on model prediction performance was firstly inspected. The modeling tasks were performed mainly on an open-source machine-learning tool, the Waikato Environment for Knowledge Analysis (WEKA) [1]. WEKA 3.8 was chosen as preset condition for *O*-glycosylated site prediction. Briefly, human *O*-glycosylated sites recorded in OGP were manually extracted as positive instances, while non-reported and non-experimental verified serine/threonine sites on *O*-glycoproteins recorded in OGP database were pretreated as negative sites (downloaded from UniProt database at 2016.06.27). Since the crude training set was highly unbalanced (positive/negative (192/8937) ≈ 1/16), high average classification accuracy but poor positive site classification accuracy was observed on the crude training set. As is shown in the confusion matrix in Table S1, the prediction precision for positive instances was only 2.127% (4/188), though the average precision is as high as 97.8928%. To solve this problem, both increasing positive instances from new records, such as multiplying positive samples (or so-called oversampling), and decreasing the proportion of negative instances (down-sampling) were tried respectively. As is shown in Figure 2B and Table S2, incorporating more O-glycosylated sites from newly published data promoted the positive prediction precision (4/188, 507/601, 1692/69) while lowering the ratio of negative to positive instances promoted the receiver operating characteristic (ROC) curve area of the classification model, which increased the robustness of prediction models. However, considering that simply multiplied positives greatly increased the risk of over fitting and was prone to larger generalization error, downsampling methods were finally adopted to improve glycosylated site prediction precision as well as keeping the stability of prediction model. As a result, a total of 1754 positive site-central sequences and 1754 negative site sequences were included as final training set.

It is worth mentioning that the prediction performance can still be improved with the extension of *O*-glycosylation records in the future to serve as a more accurate prediction tool.

**Table S2 Effect of** **scale and ratio of positives and negatives on model prediction**

| **TP ratio** | **Sampling** | **Precision** | **Confusion matrix** | | |
| --- | --- | --- | --- | --- | --- |
|  |  |  | **TP/FP** | **TN/FN** | |
| 192 + 8937 | Early version | 97.8928% | 4/188 | | 8869/68 |
| 668 + 8985 | Median version | 94.1670% | 121/547 | | 8968/16 |
| 668 + 668 | 1:1 downsampling | 75.4491% | 507/161 | | 501/167 |
| 668 + 1336 | 1:2 downsampling | 99.3995% | 3340/0 | | 8910/74 |
| 1754 + 1754 | Final version | 90.5929% | 1692/62 | | 1473/281 |

*Note*: TP, true positive; FP, False Positive; TN, True Negative; FN, False Negative.

**Optimization of sequon length**

It was reported that *O*-glycosylation is the enzymatic addition of N-acetyl galactosamine (GalNAc) to serine and threonine residues by a large family of polypeptide GalNAc-transferases (GalNAc-Ts) [2]. Therefore, amino acid (AA) sequon was expected to have great impact on whether a site can be O-glycosylated or not. In this work, sequons of 3, 4, and 5 AAs forward and backward off potential modification sites (*i.e.*, a total length of 7, 9, and 11 AAs respectively) were extracted to test their effects on model prediction performance. Deficient of AAs was complemented with letter “X”. As is shown in Figure 2C, with the number of AAs increasing, prediction precision and area under curve (AUC) slightly increased, indicating longer sequences had better performance. Taken into consideration both the fact that the catalytic domain of GalNAc transferases was about 12 AAs and the risk of increasing error rate with complements of more unnatural amino acid “X” for longer sequences, a 11 AAs length was finally used for model prediction.

**Comparison of different algorithms’ performance on site prediction**

Due to the complexity of *O*-GalNAcylated site catalytic mechanism, a variety of classification algorithms have been tested for *O*-glycosylated site prediction [3], including support vector machine (SVM) [4], artificial neural networks (ANN) [5], Naïve Bayesians (NB) [6], C4.5 decision tree (C4.5) [7], k-nearest neighborhood (KNN) [8], and random forest (RF) [9]. In this study, the performances of different classification algorithms on *O*-glycosylation site prediction were carefully surveyed to figure out the most suitable one. Parameters for each classification algorithms were well-optimized and listed as follows:

**SVM:**

weka.classifiers.functions.LibSVM -S 0 -K 2 -D 3 -G 0.0 -R 0.0 -N 0.5 -M 40.0 -C 1.0 -E 0.001 -P 0.1 -B -seed 1

with classification type set as C-SVC and kernel type set as radial basis function: exp(-gama*|u-v|^2)

**ANN:**

weka.classifiers.functions.MultilayerPerceptron -L 0.3 -M 0.2 -N 500 -V 0 -S 0 -E 20 -H a -R

with layer number set as ‘a’ = (attributes + classes) / 2.

**C4.5:**

weka.classifiers.trees.J48 -C 0.25 -M 2

**KNN:**

weka.classifiers.lazy.IBk -K 5 -W 0 –A

with the number of neighbors(KNN) optimized as 5

**RF:**

weka.classifiers.trees.RandomForest -I 500 -K 0 -S 1

with number of tree was set as 500 which was tested to be stable for the classification model in this case

All classification modeling were performed on the same training set mentioned in File S3 under a 10-fold cross validation. The performances of each algorithm were shown in Table S3, Figure 2D and E. In summary, RF outperformed other classification methods with a prediction precision of 91.5% and ROC area of 0.983 and was finally adopted as the final algorithms for *O*-glycosylation site prediction. It is currently the highest precision model for O-glycosylation site prediction.

To our understanding, there might be two reasons for the superior performance of RF over other algorithms: 1) the multi-decision characteristics of RF better mimics the multiple transferases-regulated O-glycosylation; 2) the site training set adopted in our modeling process is the largest by far so that the modeling can cover more comprehensive site pattern compared with existing methods.

**Table S3 Prediction performance of several algorithms**

| **Algorithms** | **TP rate** | **FP rate** | **Precision** | **Recall** | **F-measure** | **ROC area** | **Time cost(s)** |
| --- | --- | --- | --- | --- | --- | --- | --- |
| SVM | 0.764 | 0.236 | 0.765 | 0.764 | 0.764 | 0.842 | 7.33 |
| ANN | 0.725 | 0.275 | 0.726 | 0.725 | 0.725 | 0.804 | 5355.3 |
| NB | 0.771 | 0.229 | 0.772 | 0.771 | 0.77 | 0.856 | 0.01 |
| C4.5 | 0.734 | 0.266 | 0.734 | 0.734 | 0.734 | 0.795 | 0.05 |
| KNN | 0.724 | 0.276 | 0.732 | 0.724 | 0.722 | 0.804 | 0.01 |
| RF | 0.909 | 0.091 | 0.915 | 0.909 | 0.909 | 0.983 | 1.79 |

**References**

[1] Smith TC, Frank E. Introducing machine learning concepts with WEKA. Methods Mol Biol 2016;1418:353–78.

[2] Steentoft C, Vakhrushev SY, Joshi HJ, Kong Y, Vester-Christensen MB, Schjoldager KTBG, et al. Precision mapping of the human O-GalNAc glycoproteome through SimpleCell technology. EMBO J 2013;32:1478–88.

[3] Yang X, Han H. Factors analysis of protein O-glycosylation site prediction. Comput Biol Chem 2017;71:258–63.

[4] Chang CC, Lin CJ. LIBSVM: A library for support vector machines. ACM Trans Intell Syst Technol. 2011;2:27.

[5] Rumelhart DE, Hinton GE, Williams RJ. Learning representations by back-propagating errors. Nature. 1986;323:533–6.

[6] John GH, Langley P. Estimating continuous distributions in Bayesian classifiers. In: [Besnard](https://dl.acm.org/profile/81100211872) P, [Hanks](https://dl.acm.org/profile/81490675764) S, editors. UAI'95: proceedings of the eleventh conference on uncertainty in artificial intelligence, San Francisco: Morgan Kaufmann Publishers Inc;1995,p.338–45.

[7] Quinlan R. C4.5: Programs for Machine Learning. San Mateo: Morgan Kaufmann;1993.

[8] Aha DW, Kibler D, Albert MK. Instance-based learning algorithms. Mach Learn. 1991;6:37–66.

[9] Breiman L. Random forests. Mach Learn. 2001;45:5–32.
